# Hyperoxygenation During Mid-Neurogenesis Accelerates Cortical Development in the Fetal Mouse Brain

**DOI:** 10.3389/fcell.2022.732682

**Published:** 2022-03-17

**Authors:** Franz Markert, Alexander Storch

**Affiliations:** ^1^ Department of Neurology, University of Rostock, Rostock, Germany; ^2^ German Center for Neurodegenerative Diseases (DZNE) Rostock/Greifswald, Rostock, Germany

**Keywords:** oxygen, hyperoxia, corticogenesis, neural stem cells, apoptosis, brain development, microglia, cortical layers

## Abstract

Oxygen tension is well-known to affect cortical development. Fetal brain hyperoxygenation during mid-neurogenesis in mice (embryonic stage E14.5. to E16.5) increases brain size evoked through an increase of neuroprecursor cells. Nevertheless, it is unknown whether these effects can lead to persistent morphological changes within the highly orchestrated brain development. To shed light on this, we used our model of controlled fetal brain hyperoxygenation in time-pregnant C57BL/6J mice housed in a chamber with 75% atmospheric oxygen from E14.5 to E16.5 and analyzed the brains from E14.5, E16.5, P0.5, and P3.5 mouse embryos and pups *via* immunofluorescence staining. Mid-neurogenesis hyperoxygenation led to an acceleration of cortical development by temporal expansion of the cortical plate with increased NeuN^+^ neuron counts in hyperoxic brains only until birth. More specifically, the number of Ctip2^+^ cortical layer 5 (L5) neurons was increased at E16.5 and at birth in hyperoxic brains but normalized in the early postnatal stage (P3.5). The absence of cleaved caspase 3 within the extended Ctip2^+^ L5 cell population largely excluded apoptosis as a major compensatory mechanism. Timed BrdU/EdU analyses likewise rule out a feedback mechanism. The normalization was, on the contrary, accompanied by an increase of active microglia within L5 targeting Ctip2^+^ neurons without any signs of apoptosis. Together, hyperoxygenation during mid-neurogenesis phase of fetal brain development provoked a specific transient overshoot of cortical L5 neurons leading to an accelerated cortical development without detectable persistent changes. These observations provide insight into cortical and L5 brain development.

## Introduction

Oxygen tension during development is known to critically affect brain development (Fagel et al., 2006; Schneider et al., 2012; Porzionato et al., 2015; Wagenfuhr et al., 2015; Lange et al., 2016; Wagenfuhr et al., 2016). Thereby, the effects depend on timing and intensity of oxygen application: while short-term hyperoxygenation is able to enhance neurogenesis and brain size, chronic hyperoxygenation can lead to adverse effects (Wagenfuhr et al., 2015; Wagenfuhr et al., 2016; Markert et al., 2020). Indeed, short-term hyperoxygenation during mid-neurogenesis of fetal mouse brain development (embryonic stages E14.5 to E16.5) leads to an immediate expansion of a distinct proliferative cell population basal of the subventricular zone (SVZ) constituting a new neurogenic cell layer similar to the outer SVZ (OSVZ), which contributes to corticogenesis by heading for deeper cortical layers as a part of the cortical plate (CP) (Wagenfuhr et al., 2015). Finally, the number of Ctip2^+^ neurons in the deeper layer 5 (L5) of the CP projecting into various brain regions is markedly increased (Hattox and Nelson 2007; Oswald et al., 2013; Wagenfuhr et al., 2015). This phenomenon is of high interest, since alterations within the cortical L5 cell population are directly linked to diseases such as schizophrenia (Kolomeets and Uranova 2019; Mi et al., 2019), which is also linked with oxidative stress and various other factors during development (Chew et al., 2013; Górny et al., 2020). Despite these known effects of maternal hyperoxygenation with subsequent changes of the oxygen tension of brain tissue *in utero*, a pilot study applying maternal oxygenation in humans, although in a more chronic treatment scheme, shows initial morphological changes of the head, but no differences in neurodevelopmental testing of the children (Edwards et al., 2018). These results raise not only concerns about the safety of maternal hyperoxygenation therapy (Rudolph 2020) but also the questions whether and how the brain is able to better compensate for changes of neuronal plasticity during development to normalize the cortical structure.

The process of embryonic/fetal brain development is highly orchestrated through main events like proliferation, differentiation, and migration of neuronal stem cells (Noctor et al., 2001; Talamillo et al., 2003; Haubensak et al., 2004; Noctor et al., 2004) and morphological and functional shaping of cortical cell populations (White and Barone 2001; Blanquie et al., 2017). Thereby, radial glia cells located at the ventricular surface develop into cortical neurons through the Pax6, Tbr2, and Tbr1 axis where the resulting cells migrate through the cortex and form the cortical inside-out layering (Englund et al., 2005; Agirman et al., 2017). The resulting number of neurons seems to be prenatally regulated through invading microglia capable of phagocytizing and controlling the number Pax6^+^ or Tbr2^+^ cells (Cunningham et al., 2013). Other suggested mechanisms include a feedback signal from cortical deep layer cells to the radial glia affecting the generation of upper layer cells as well as already occurring apoptosis of neuroprecursor cells (Blaschke et al., 1996; Toma et al., 2014). After birth, apoptosis of postmitotic neurons particularly becomes prominent in the cortex where around 50% of all neurons die (Dekkers et al., 2013; Wong and Marín 2019). This mechanism likely regulates the number of cortical neurons in an area-dependent manner through their electrical activity and indicates a specific postnatal connectivity control (Blanquie et al., 2017).

Although the short-term effects of hyperoxygenation during mid-neurogenesis of fetal mouse brain development with immediately enhanced neurogenesis particularly within cortical L5 are reported, the subsequent consequences of these phenomena during later cortical development remain enigmatic. We therefore used our established model of maternal hyperoxygenation to investigate the effects of increased oxygen tension during mid-neurogenesis (E14.5–E16.5) on later cortical development until the early postnatal state (Wagenfuhr et al., 2015). Moreover, the model allows the investigation of potential mechanisms mediating the reshape of the cortical structure during late fetal and early postnatal cortical development.

## Materials and Methods

### Animals and Oxygen Treatment

C57BL/6J timed-pregnant mice were housed in their home cages within a preconditioned oxygen chamber (InerTec, Grenchen, Switzerland) at 75% oxygen or room air (21% oxygen; control condition). During the whole treatment protocol, all animals were handled by the same investigator. Fetuses of both groups showed an ordinary morphology. Pregnant mice for analysis of postnatal day 0.5 (P0.5) fetuses intraperitoneally received BrdU (50 mg/kg body weight) at E14.5, the start of hyperoxia treatment, and EdU (25 mg/kg body weight) at E17.5, 1 day after the end of the oxygen treatment. All data were gathered from randomly chosen embryos or pups from at least three independent litters per group. All animals were maintained and treated with permission of the local Department of Animal Welfare (Landesamt für Landwirtschaft, Lebensmittelsicherheit und Fischerei Mecklenburg-Vorpommern) (reference number 7221.3-1-043/16) and comply with the Tierschutzgesetz and Verordnung zur Umsetzung der Richtlinie 2010/63/EU from Germany. Of note, our study was initially designed including a hypoxia group (10% oxygen), but it was not possible to gather data for postnatal maternal hypoxia group as the mice tend to infanticide. To secure animal welfare and to be in line with German law, we had to cancel these experiments.

The brains of embryos and pups were dissected prior and immediately after oxygen treatment [E14.5 (for estimating the cortical volume) and E16.5] and postnatally at P0.5 and P3.5, fixed for 24 h in 4% paraformaldehyde (Merck, Darmstadt, Germany), and kept in 30% sucrose (Carl Roth, Karlsruhe, Germany) in DPBS (Thermo Fisher Scientific, Waltham, United States). Then brains were snap-frozen, sectioned coronal at 20-µm thickness using a cryomicrotome (Leica Biosystems, Nussloch, Germany), and mounted on Superfrost Plus slides (Thermo Fisher Scientific). The slides were stored at 4°C until staining.

### Immunofluorescence

Slides were washed with wash buffer (Agilent, Santa Clara, United States), and heat-induced antigen retrieval was performed using 10 mM sodium citrate (Carl Roth) with 0.05% Tween 20 (SERVA, Heidelberg, Germany) for 30 min at 95°C or 2 N HCl for 30 min at 37°C for BrdU staining. After 20 min at room temperature, slides were washed with Tris buffered saline/Tween 20 (TBST), treated with TBST containing 0.2% Triton X-100 (Carl Roth) and 10% donkey serum (Merck) for 30 min, and were incubated with primary antibodies overnight at 4°C. The following primary antibodies were used: rabbit anti-NeuN (Merck, ABN78, RRID: AB_10807945), chicken anti-NeuN (Merck, ABN91, RRID: AB_11205760), rabbit anti-cleaved-caspase-3 (CC3; Cell Signaling, 9661S, RRID: AB_2341188), mouse anti-BrdU (Thermo Fisher Scientific, B35128, RRID: AB_2536432), rabbit anti-Tbr1 (Abcam, ab31940, RRID: AB_2200219), mouse anti-Satb2 (Abcam, ab51502, RRID: AB_882455), rat anti-Ctip2 (Abcam, ab18465, RRID: AB_2064130), rabbit anti-Ctip2 (Abcam, ab28448, AB_1140055), rabbit anti-Iba1 (Wako, 019-19741, RRID: AB_839504), goat anti-Iba1 (Abcam, ab5076, RRID: AB_2224402), rabbit anti-CD68 (Abcam, ab125212,RRID: AB_10975465), rat anti-CD68 (BioRad, MCA1957GA, AB_324217), or guinea pig anti-vGluT2 (Merck, AB2251-I, RRID: AB_1587626). Subsequently, slides were incubated with corresponding secondary antibodies (Thermo Fisher Scientific), and nuclei were stained with Hoechst 33258 (Merck). For EdU analysis, slides were stained using Click-iT staining kit (Thermo Fisher Scientific) as described by the manufacturer. Finally, slides were mounted with Fluoromount-G (Biozol, Eching, Germany).

### Imaging and Measurements

Most images were taken with AxioObserver Z1 with Apotome using ZEN blue 2.3 software with Tiles and Position module (all from Carl Zeiss, Oberkochen, Germany). Z-stack images of microglia-targeting Ctip2^+^ cells were taken with LSM900 with Airyscan (Carl Zeiss). Hoechst images of every sixth section with a thickness of 20 µm were taken with ×2.5 objective and subsequently used for determining the volume of the whole brain and the CP corresponding to the mouse brain atlas (Allen-Institute, 2008). Thereby, we used the corpus callosum and the lateral ventricles for orientation dorsal/between the hemispheres and for lateral the piriform region and the endopiriform nucleus. For caudal sections, we used the thinner subiculum layer in the extension of hippocampal C1 layer (excluded) for dorsal orientation (Supplementary Figure S1). The volume was calculated by adding up the data of each slice (midpoint type) and then multiplying by 120 (every sixth slice of 20-µm thickness).

For analysis of apoptosis, fluorescence images of the whole hemispheres from the developing parietal cortex areas were taken. CC3^+^ cells were counted with ZEN blue 2.3 in the cortex from the dorsal to ventral site corresponding to the area described above. For double staining, CC3^+^ were searched as described above and imaged as Z-stack with Apotome mode. For specific marker analysis, at least four images of the cortex were taken as Z-stack with 1-µm steps. The corresponding cells or VGlut2^+^ synapses were counted in the middle focal plane using either ZEN analysis software (Tbr1, Ctip2, and Satb2) or ImageJ (BrdU, EdU, and NeuN). L5 neurons at postnatal stages were defined by high Ctip2 expression levels as described by McKenna et al. (2011). Iba1^+^ cells and double/triple-stained cells were manually counted through the Z-stacks within the middle cortical sections in the rostro-caudal axis by the same investigator who was blinded for the treatment groups.

### Statistics

All statistical analyses were performed with RGUI 3.4.1 (R Foundation for Statistical Computing, Vienna, Austria) or SPSS version 25.0 (SPSS Inc., Chicago, IL). If not otherwise stated, statistical significance was evaluated by unpaired two-sided *t*-test or two-way ANCOVA followed by pairwise *t*-test including Bonferroni correction. Cortical volume was analyzed independent of the developmental state due to its well-known physiological large volume expansion after birth. The numbers of analyzed embryos and pups gathered from at least three independent litters are indicated by “*n*.” All data are displayed as means ± s.e.m. with the numbers of analyzed embryos and pups indicated for each experiment. The significance level was set to *p* < 0.05 (two-tailed test).

## Results

### Oxygen-Induced Cortical Expansion During Fetal Brain Development is Equalized at Early Postnatal Stage

**FIGURE 1:**
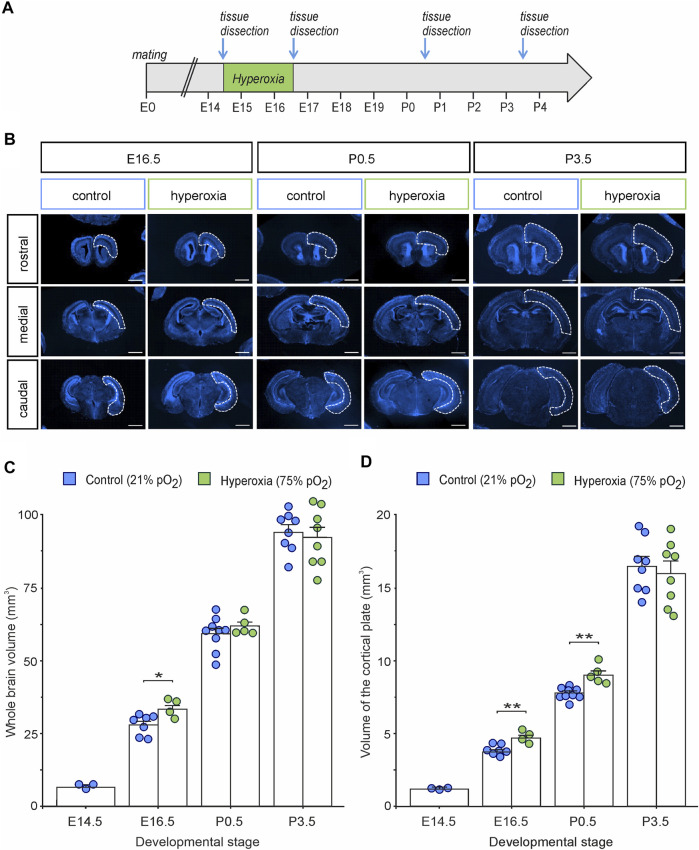
The effects of fetal brain hyperoxia during mid-neurogenesis (E14.5 to E16.5) on volume of the cortical plate (CP) during later brain development in mice. **(A)** Experimental treatment scheme for fetal brain hyperoxygenation (by maternal application of 75% O_2_) during mid-neurogenesis (E14.5 to E16.5). **(B)** Representative fluorescence images from rostral, middle, and caudal slices of E16.5, P0.5, and P3.5 brains. Slices were stained with Hoechst (blue). Dashed lines outline the cortical area used to estimate cortical volume. Scale bars, 1,000 µm. **(C,D)** Quantification of whole-brain volume **(C)** and the volume of the CP **(D)** showed increased cortical volume in hyperoxic brains at E16.5 and P0.5 (only CP) but not at P3.5 as compared to normoxic controls. Note that the value at E14.5 serves as starting point of volume just before the hyperoxic treatment, and thus, no hyperoxic condition was tested. Data are means ± s.e.m. [E14: *n* = 3; E16.5: *n* = 7 (control), *n* = 4 (hyperoxia); P0.5: *n* = 9 (control), *n* = 5 (hyperoxia); P3.5: *n* = 8 (control and hyperoxia)]. *p < 0.05 and **p < 0.01 from unpaired two-sided *t*-tests (non-significant comparisons are not marked for clarity).

To assess the time course and putative long-term persistency of the effects of oxygen tension on the rapidly changing and highly regulated fetal cortical development, we applied the already introduced mouse model of maternal hyperoxygenation known to reliably control tissue oxygen tension and subsequently neurogenesis within the developing fetal mouse brain (Wagenfuhr et al., 2015; Wagenfuhr et al., 2016). We thus applied maternal hyperoxygenation to time-pregnant mice at mid-neurogenesis from embryonic stage E14.5 to E16.5 and investigated cortical morphology during embryonic development from E14.5 to early postnatal stage at P3.5 (Figure 1A). The hyperoxia and control groups showed no abnormalities in their spontaneous or litter care behavior. All embryos and pups displayed normal morphology at all developmental stages examined, but the brains of the hyperoxia group appeared visually and quantitatively increased in their size at E16.5, but not on other developmental stages (Figures 1B,C). Indeed, mouse embryos of the hyperoxia group showed a 1.2-fold increase in the volume of the CP as compared with normoxic controls at E16.5 (*p* = 0.002), which persisted until birth (P0.5; *p* = 0.015), but not until P3.5 (*p* = 0.653; unpaired two-sided *t*-test; *n* = 4–9; Figure 1D).

### Hyperoxygenation During Mid-Neurogenesis Accelerates But Does Not Increase Cortical Neurogenesis

**FIGURE 2:**
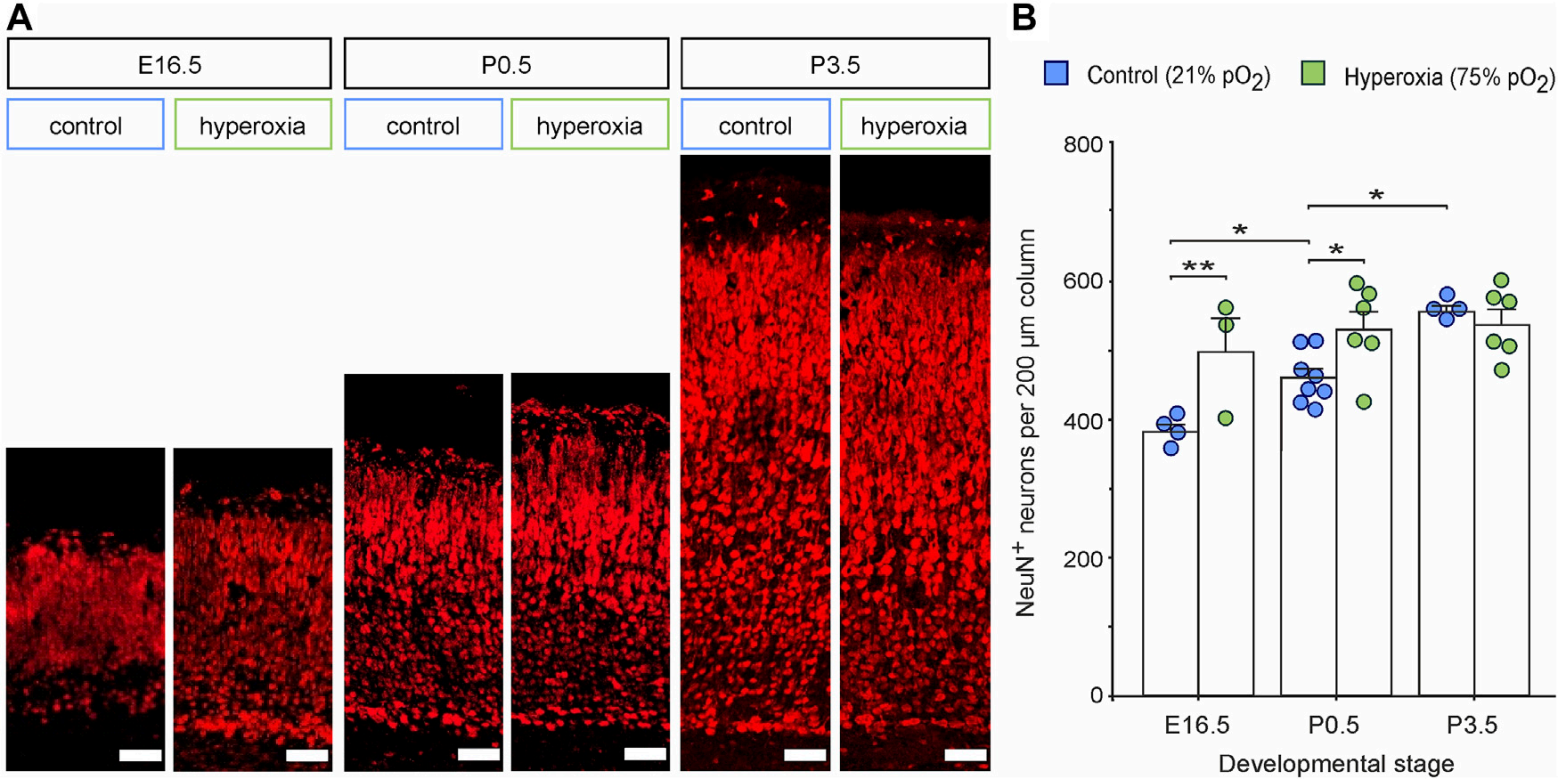
The effects of fetal brain hyperoxia during mid-neurogenesis (E14.5 to E16.5) on the numbers of cortical neurons during later brain development in mice. **(A)** Representative fluorescence images from the CP in the middle sections along the rostro-caudal axis stained with NeuN (red). Scale bars, 50 µm. **(B)** Quantification of NeuN^+^ cortical neurons in the brain slices. Data are means ± s.e.m. [E16.5: *n* = 4 (control), *n* = 3 (hyperoxia); P0.5: *n* = 8 (control), *n* = 6 (hyperoxia); P3.5: *n* = 4 (control), *n* = 6 (hyperoxia)]. *p < 0.05 and **p < 0.01 from two-way ANOVA with *post-hoc* two-sided *t*-test with Bonferroni correction (non-significant comparisons are not marked for clarity). For full statistics for **(B)**, see Supplementary Table S1.

To further evaluate cortical development, we used NeuN staining and analyzed the number of neurons within the middle cortical sections along the rostro-caudal axis (Figure 2). The hyperoxic embryos showed an increase of NeuN^+^ neurons per volume at E16.5 with a 1.3-fold higher neuronal density as compared with normoxic control animals (*p* = 0.005), which persisted until birth (P0.5) with a still 1.2-fold higher neuronal density (*p* = 0.013), but not until P3.5 (*p* = 0.581, all from *post hoc* two-sided *t*-test with Bonferroni adjustment; Figure 2B). The neuronal density in the control group rose continuously between E16.5 and P3.5, while in the hyperoxia group, the maximum neuronal density seems to be already reached at earlier developmental stages at E16.5 (Figure 2B). Of note, neuronal density in the hyperoxia group never exceeded that in the control group, indicating an accelerated but not increased cortical neurogenesis (Figure 2B).

### Postnatal Cortical Normalization Occurs in a Layer-Specific Manner

**FIGURE 3:**
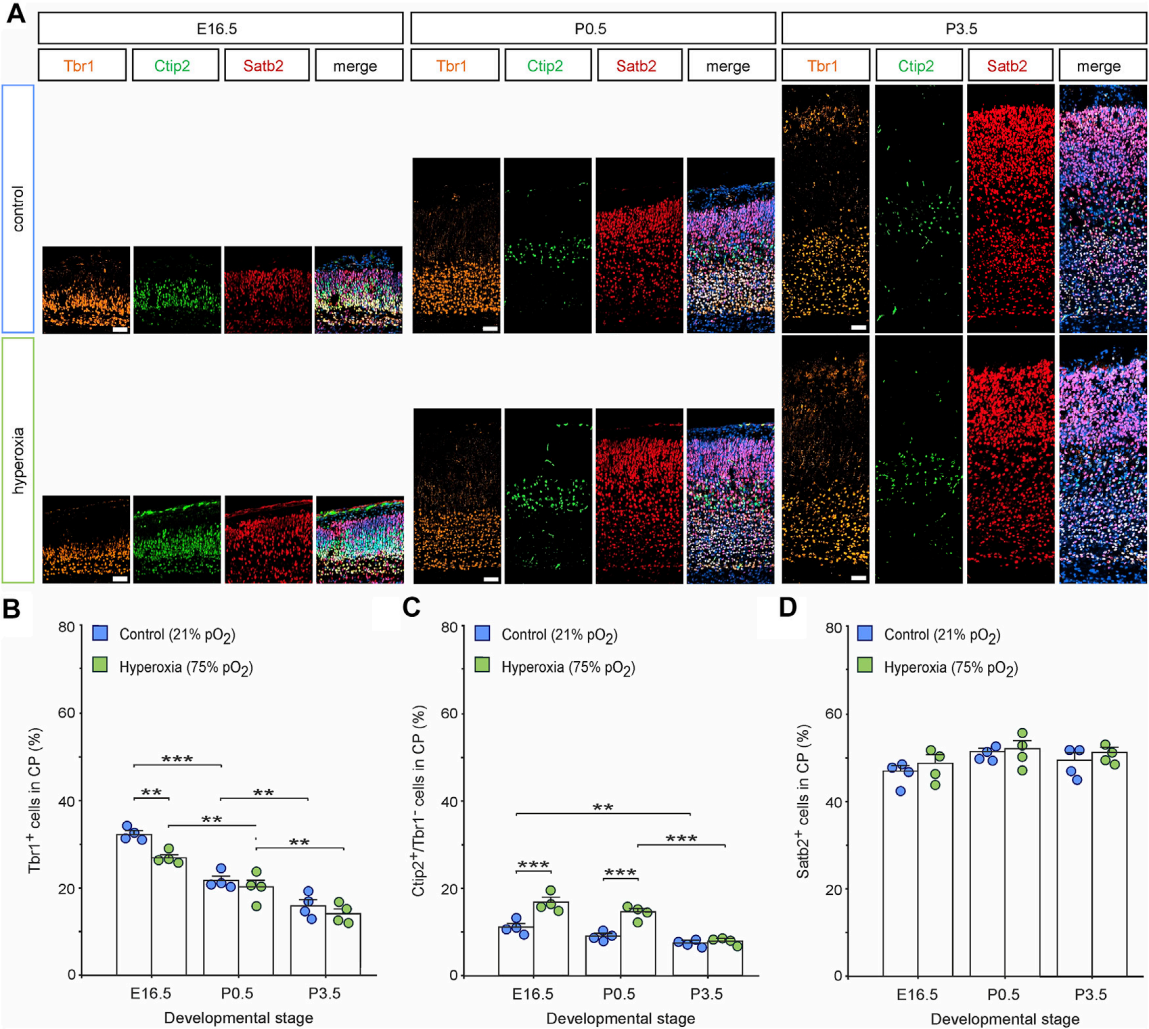
The effects of fetal brain hyperoxygenation on layer-specific distribution of neurons. **(A)** Representative fluorescent images of Tbr1^+^ (orange), Ctip2^+^ cells (green), and Satb2^+^ cells (red) from E16.5, P0.5, and P3.5 in the middle cortical sections along the rostro-caudal axis of hyperoxic and normoxic control mice. Hoechst (blue) was used to stain cell nuclei shown in the merged image. Scale bars, 50 µm. **(B–D)** Quantification of the distribution of Tbr1^+^ cortical subplate/layer 6 neurons **(B)**, Ctip2^+^/Tbr1^−^ layer 5 neurons **(C)**, and Satb2^+^ upper layer-specific neurons **(D)** showed a specific increase of layer 5 neurons at E16.5 and P0.5 but not at P3.5. Data are means ± s.e.m. (*n* = 4 for all animal groups). **p < 0.01 and ***p < 0.001 from two-way ANOVA with *post-hoc* two-sided *t*-test with Bonferroni correction (non-significant comparisons are not marked for clarity). For full statistics, see Supplementary Tables S2–S4.

Whether the equalization of cortical volume and neuronal density at birth after fetal hyperoxygenation during the mid-neurogenesis phase is capable of functioning as a control mechanism for regulating neuronal layer specificity and neural circuits or whether it originates from increased raw cell numbers competitive to each other remains elusive. To shed light on this aspect, we performed a layer-specific analysis using a panel of markers Tbr1, Ctip2, and Satb2 of hyperoxic mouse embryos and pups through the developmental stages E16.5, P0.5, and P3.5 and performed a quantitative analysis for Tbr1^+^ cells as characteristic for neurons of the SP and L6 (Hevner et al., 2001), Ctip2^+^/Tbr1^−^ cells representing L5 neurons (Arlotta et al., 2005), and Satb2^+^ cells as neurons of the upper cortical layers (Figure 3) (Britanova et al., 2008). The number of Tbr1^+^ cells in the CP decreased continuously from E16.5 to P3.5 (Figure 3B)**.** Of note, the percentage of Tbr1^+^ cells in the CP was reduced at E16.5, although the total number of Tbr1^+^ cells was not affected (Figure 3B; Supplementary Figure S2).

Quantification of the percentage of L5-specific neurons (Ctip2^+^/Tbr1^−^ cells; (Hevner et al., 2001; Arlotta et al., 2005) showed a persistent increase within the hyperoxic group at E16.5 (1.5-fold) and P0.5 (1.6-fold; Figure 3C), which further supports our previous data and even further demonstrates that fetal brain hyperoxia evoked persistent effects on L5 (Wagenfuhr et al., 2015). The same layer marker panel revealed no differences in the number of Ctip2^+^ cells at P3.5. Comparing the time course of Ctip2^+^ cell numbers during cortical development, there was a slow drop of the percentage of Ctip2^+^ cells within the CP between E16.5 and P3.5 in normoxic mice, but a later drop just after birth in hyperoxic mice, suggesting that the specific rearrangement of cortical L5 is postponed by hyperoxia (Figure 3C). We also found an increase in the absolute numbers of L5 neurons between E16.5 and P0.5. Since no more L5 neurons are generated at this time, we assume that this represents a change in the expression of Ctip2 rather than ongoing neurogenesis (McKenna et al., 2011; Toma et al., 2014).

The number of upper-layer Satb2^+^ neurons was not affected by hyperoxia through all developmental stages (Figure 3D; Supplementary Figure S2).

### Microglia are Involved in Postnatal Cortical Normalization After Fetal Hyperoxygenation

The postnatal regulation of the CP after fetal hyperoxygenation prompted us to evaluate the possible underlying mechanisms. There are already known mechanisms capable of controlling the number of brain cells including neurons during development (Cunningham et al., 2013; Toma et al., 2014; Blanquie et al., 2017): apoptosis known to occur during early postnatal cortical development; feedback mechanisms where the increased number of neurons signals a feedback to neuronal stem cells or microglia able to phagocytose brain cells. We consequently analyzed Iba1 staining for the number of microglia, CC3 staining as an established marker for apoptosis, and time-delayed BrdU/EdU labelling for estimating the birthdate of the resulting neurons during brain development.

**FIGURE 4:**
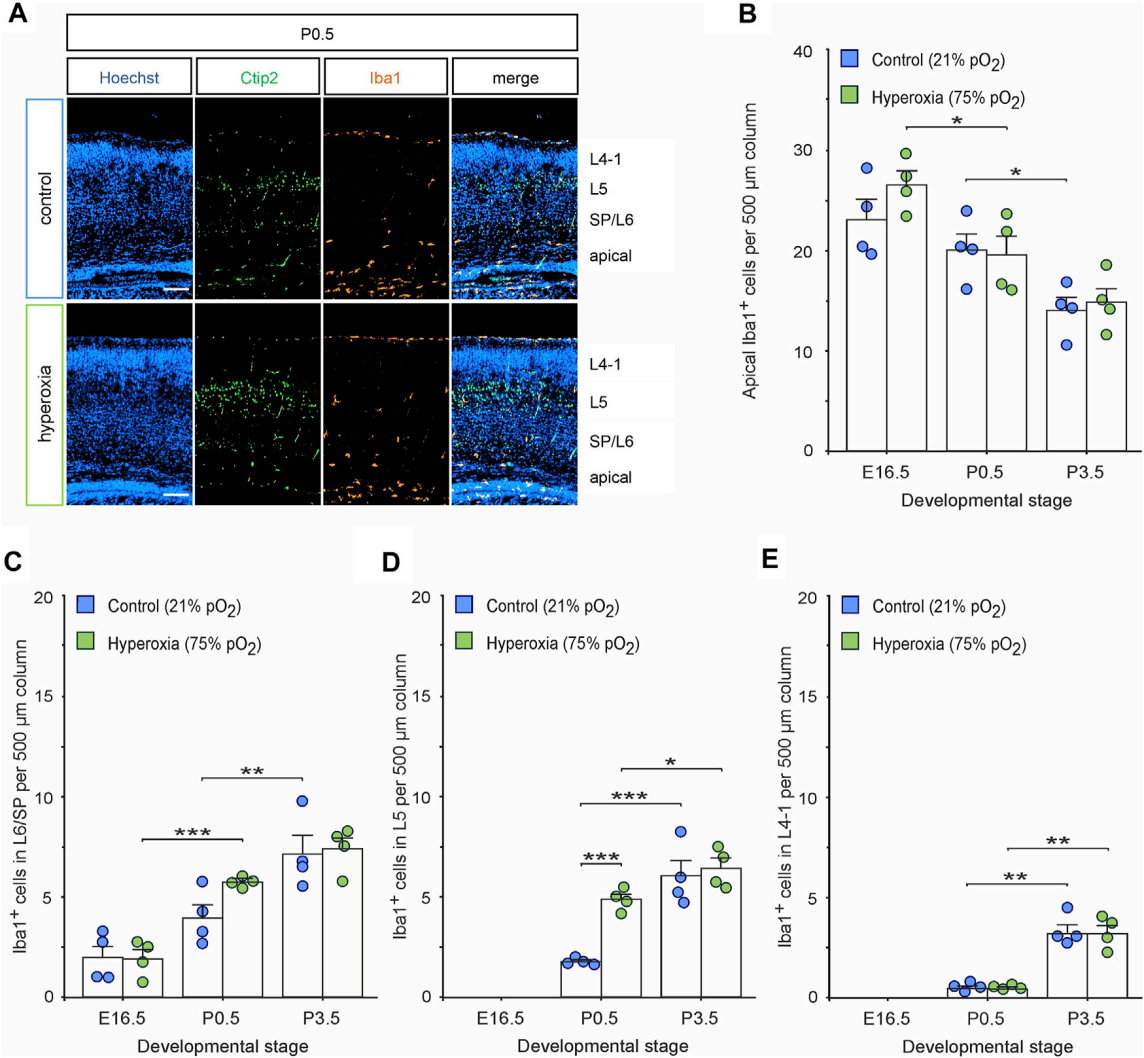
The effects of hyperoxygenation on microglial distribution within the developing cortex. **(A)** Representative fluorescent images of Iba1^+^ cells (orange) from P0.5 in the middle cortical sections along the rostro-caudal axis of hyperoxia-treated and control mice. Ctip2^+^ (green) was used for layer determination, and Hoechst (blue) was used to stain cell nuclei. Scale bars represent 100 µm. **(B–E)** Quantification of Iba1^+^ apical microglia **(B)**, within subplate/layer 6 (SP/L6) **(C)**, layer 5 (L5) **(D)**, and upper layers 4-1 (L4-1) **(E)** showed an increase of microglia within L5 at P0.5. Data are means ± s.e.m. (*n* = 4 for all animal groups). *p < 0.05, **p < 0.01, and ***p < 0.001 from two-way ANOVA with *post-hoc* two-sided *t*-test with Bonferroni correction (non-significant comparisons are not marked for clarity). For full statistics, see Supplementary Tables S5–S8.

**FIGURE 5:**
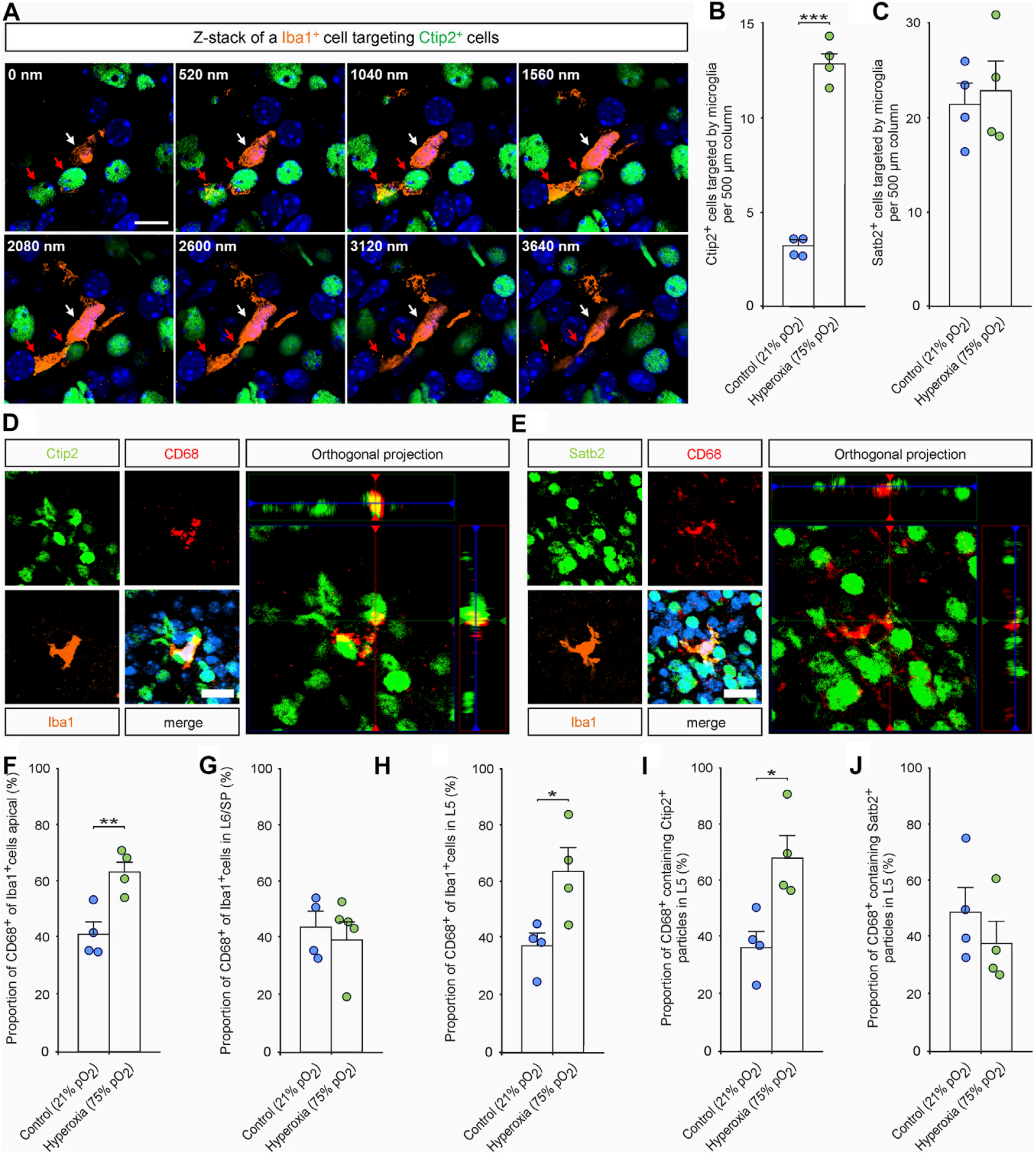
The effects of hyperoxygenation on the layer-specific activation of microglia and their targeting and phagocytosis of neurons within the developing cortex. **(A)** Representative Z-stack images of an Iba1^+^ microglia cell (white arrow) targeting Ctip2^+^ cells (red arrows) in layer 5 of a P0.5 mouse cortex. Scale bars represent 10 µm. **(B,C)** Quantification of Ctip2^+^ cells targeted by Iba1^+^ microglia **(B)** and Satb2^+^ cells targeted by Iba1^+^ microglia **(C)** at P0.5. Data are means ± s.e.m. (*n* = 4). ***p < 0.001 from unpaired two-sided *t*-test (non-significant comparison is not marked for clarity). **(D,E)** Representative fluorescent images of triple staining with Iba1, CD68, and Ctip2 **(D)** or Satb2 **(E)** in the middle cortical sections along the rostro-caudal axis of hyperoxia-treated and control mice at P0.5. Orthogonal projections of CD68 and Ctip2/Satb2 show co-localization of the markers. Hoechst (blue) was used to stain cell nuclei. Scale bars represent 20 µm. **(F–H)** Quantification of CD68^+^ microglia revealed an increase of active apical microglia **(F)** and L5 **(G)**, but not in L6/SP **(H)**. *p < 0.05, **p < 0.01, and ***p < 0.001 from unpaired two-sided *t*-test (non-significant comparisons are not marked for clarity; *n* = 4). **(I,J)** Quantification of CD68^+^ microglia containing neuronal markers revealed an increase of microglia containing Ctip2 particles in hyperoxia-treated mice **(I)** while there is no difference regarding contained Satb2 particles **(J)**. *p < 0.05, **p < 0.01 from unpaired two-sided *t*-test (non-significant comparisons are not marked for clarity; *n* = 4).

Immunohistochemical staining of Iba1^+^ cells labelling resting and activated microglia (Imai et al., 1996; Morgan et al., 2010) revealed an already visually detectable increase in microglia residing in L5 of hyperoxic P0.5 mouse pups (Figure 4A), which was not present in E16.5 or P3.5 brains (Supplementary Figure S3). Quantification of these Iba1^+^ cells revealed a specific 2.7-fold increase of microglial cells in L5 of hyperoxic mice (*p* < 0.001, *post hoc* two-sided *t*-test with Bonferroni adjustment), while the total number of Iba1^+^ cells, other layers, and developmental stages was not affected by oxygen (Figures 4B–E; Supplementary Figure S4). Microglia within the CP were predominantly found at and after P0.5, but the invasion specifically of L5 is accelerated, although not exceeding the number of microglia at P3.5. This is further supported by analysis of the apical and SP/L6 microglia, where the decrease of apical microglia and the successive increase of SP/L6 occurred between P0.5 and P3.5 in normoxic control mice, but already occurred between E16.5 and P0.5 in hyperoxic mice (the increase in Iba1^+^ microglia in SP/L6 at P0.5 represents only a non-significant trend). Intriguingly, we could show that microglia in L5-targeted Ctip2^+^ cells without morphological signs of apoptosis (Figure 5A). An analysis of Ctip2^+^ and Satb2^+^ cells targeted by microglia at time point P0.5 showed that Ctip2^+^ cells were targeted by microglia significantly more often in the hyperoxia group than in the control group (Figure 5B). At the same time, there was no difference in the number of Satb2^+^ cells targeted by microglia (Figure 5C; Supplementary Figure S5). We consequently analyzed the number of active microglia by using the combination of Iba1 and the microglia activation marker CD68 (Rabinowitz and Gordon 1991; Imai et al., 1996; Morgan et al., 2010; Jurga et al., 2020). Quantification revealed that there are indeed more active microglia in L5 and also apical, but not between these in L6/SP (Figures 5D–H). Further analyses revealed Ctip2^+^ and Satb2^+^ particles in these active microglia in L5, but—interestingly—only the number of active microglia containing Ctip2^+^ particles was increased in hyperoxic as compared to control brains (Figure 5I,J).

**FIGURE 6:**
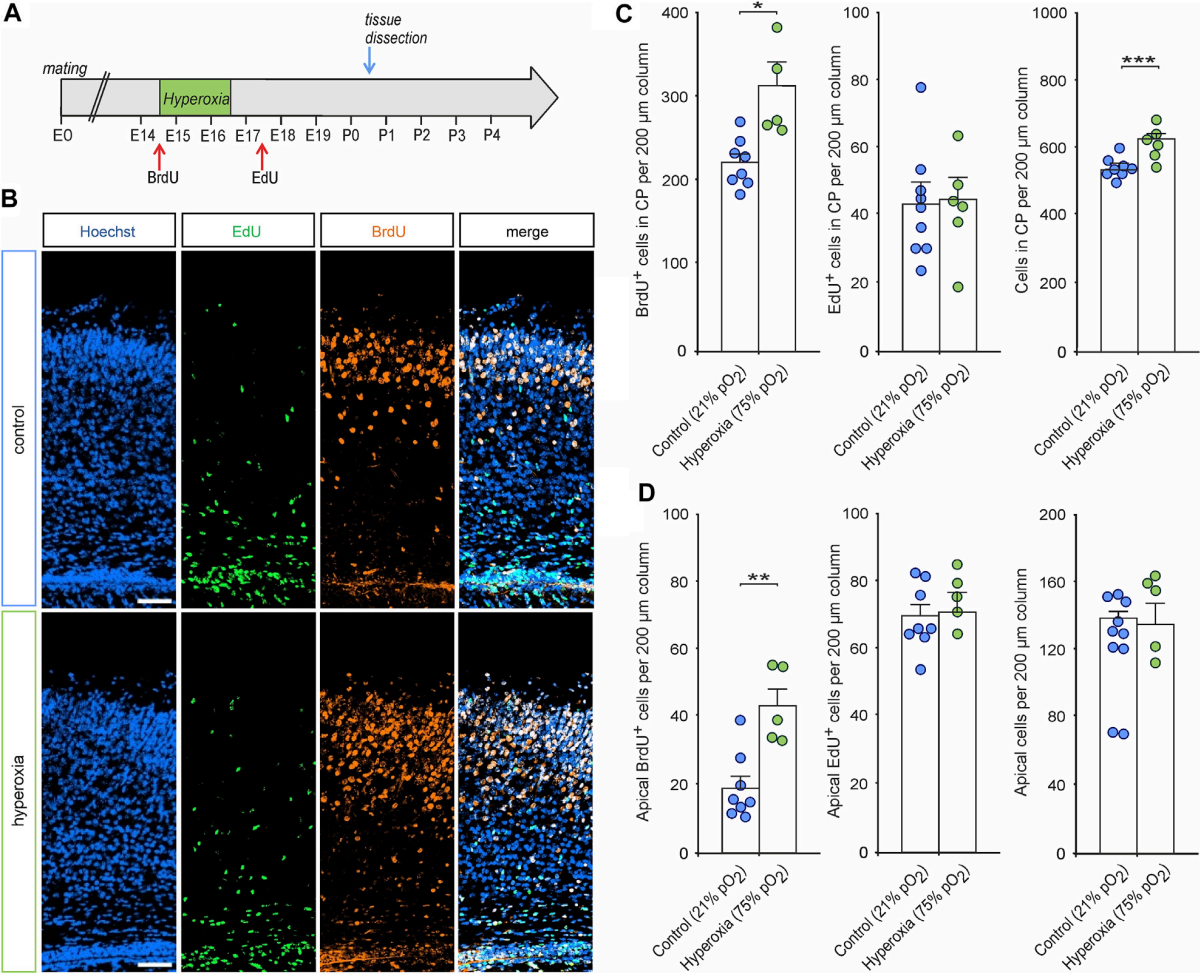
The effects of hyperoxia on cell proliferation during and after oxygen treatment. **(A)** Experimental treatment scheme for BrdU/EdU application in fetal brain hyperoxygenation during mid-neurogenesis (E14.5 to E16.5). Mice received an intraperitoneal injection with BrdU immediately before oxygen treatment and with EdU 1 day after treatment (E17.5) and subsequently analyzed at P0.5. **(B)** Representative fluorescent images of EdU^+^ (green) and BrdU^+^ cells (orange) from P0.5 in the middle cortical sections along the rostro-caudal axis of hyperoxia-treated and control mice. Hoechst (blue) was used to stain cell nuclei. Scale bars represent 50 µm. **(C,D)** Quantification of BrdU^+^, EdU^+^, and overall cells showed an increased number of CP neurons born during hyperoxia (BrdU^+^ cells) but not after hyperoxia treatment (EdU^+^ cells) within the CP **(C)** and the apical cortex **(D)** of P0.5 mouse pups. *p < 0.05, **p < 0.01, and ***p < 0.001 from unpaired two-sided *t*-test [non-significant comparisons are not marked for clarity; *n* = 8 (control) and *n* = 5 (hyperoxia)].

To further evaluate whether adaptive mechanisms contribute to normalization of the number of neurons cortex after fetal hyperoxygenation, we performed a birth-dating analysis using BrdU application immediately before and EdU application 1 day after oxygen treatment and subsequent histological analyses of BrdU/EdU incorporation by cortical cells at P0.5 (see Figure 6A for experimental paradigm). Immunostaining of both markers showed that a large proportion of CP neurons were generated at E14.5 (BrdU^+^), while cells generated at E17.5 (EdU^+^) represent a much smaller part of the developing cortex (Figures 6B,C). Quantification of BrdU^+^/EdU^+^ cells in the CP revealed a significantly 1.4-fold increase in the number of BrdU^+^ cells (*p* = 0.021) in response to hyperoxia, but not of EdU^+^ cells (*p* = 0.886, both from unpaired two-sided *t*-test), as a putative later feedback reaction. Thereby, the increased rate of BrdU^+^ cells led to an increase of the overall cells in the CP. Notably, there were more BrdU^+^ cells left at the apical side of the cortex (*p* = 0.002), but again, no change in EdU^+^ cells (*p* = 0.791, both from unpaired two-sided *t*-test; Figure 6D).

**FIGURE 7:**
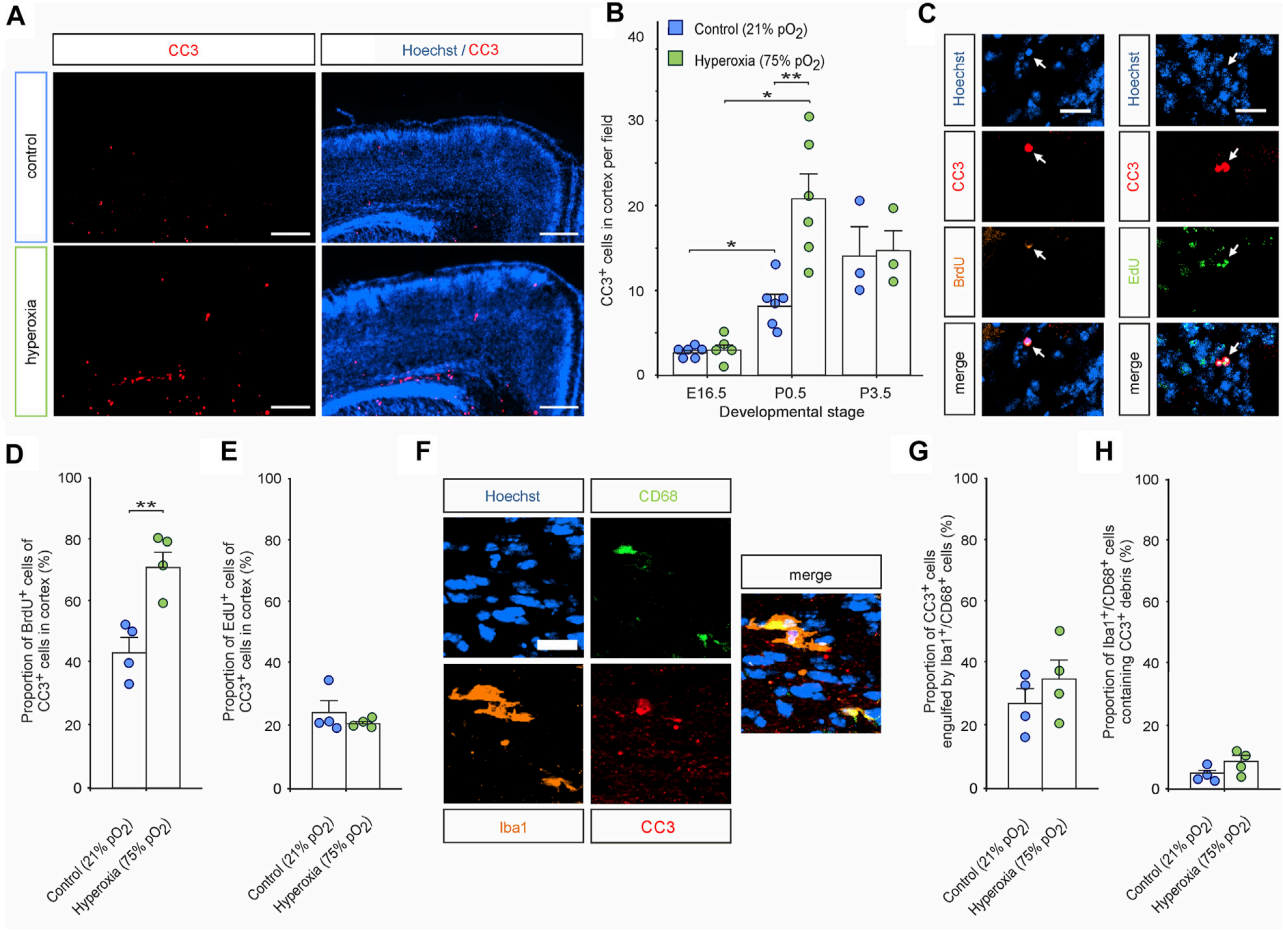
The effects of hyperoxia on apoptosis in P0.5 mice cortex after mid-neurogenesis hyperoxygenation. **(A)** Representative fluorescent images of CC3^+^ apoptotic cells (red) from P0.5 in the middle sections along the rostro-caudal axis of hyperoxia-treated and control mice. Hoechst (blue) was used to stain cell nuclei. Scale bars represent 200 µm. **(B)** Quantification of cortical CC3^+^ cells shows increased apoptosis in brain slices of hyperoxia-treated mouse embryos at P0.5 but not at E16.5 or P3.5. Data are means ± s.e.m. [E16.5: *n* = 6 (control), *n* = 5 (hyperoxia); P0.5: *n* = 6; P3.5: *n* = 3]. *p < 0.05 and **p < 0.01 from robust ANOVA with *post*-*hoc* two-sided unpaired Wilcoxon test with Bonferroni correction. For full statistics, see Supplementary Table S9. **(C)** Representative fluorescent images of BrdU^+^ (orange) and EdU^+^ (green) cells double stained with CC3 in the middle cortical sections along the rostro-caudal axis of hyperoxia-treated and control mice at P0.5. Hoechst (blue) was used to stain cell nuclei. Scale bars represent 20 µm. **(D,E)** Quantification revealed that most of the apoptotic cells are BrdU^+^ in the hyperoxia group, but not in controls **(D)**, and no differences were observed regarding apoptotic EdU^+^ cells **(E)**. **p < 0.01 from unpaired two-sided *t*-test (non-significant comparisons are not marked for clarity; *n* = 4). **(F)** Representative fluorescent image of Iba1^+^/CD68^+^ active microglia (orange/green) containing apoptotic cell debris (CC3^+^, red) in the middle sections along the rostro-caudal axis of hyperoxia-treated mice at P0.5. Hoechst (blue) was used to stain cell nuclei. Scale bars represent 20 µm. **(G,H)** Quantification of neither the percentage of apoptotic cells engulfed by microglia **(G)** nor the number microglia engulfing apoptotic cells **(H)** revealed any differences between hyperoxia-treated vs control mice (*n* = 4).

We further analyzed CC3^+^ apoptotic cells within the middle cortical sections along the rostro-caudal axis showing increased apoptosis in hyperoxic P0.5 mouse pups. Immunostaining with quantification showed that the overall number of CC3^+^ cells in the hyperoxic group is increased by 2.6-fold with apoptosis predominantly occurring in the apical regions (Figures 7A,B). We then evaluated whether layer 5 cells of the cortex were apoptotic through double labelling of CC3^+^ and Ctip2^+^, but there was almost no cell possessing both markers (<0.1% of Ctip2^+^ cells). Since apoptosis occurred mainly apical where increased proliferation could be detected in the hyperoxia group at E14.5, we analyzed the number of BrdU^+^/CC3^+^ and EdU^+^/CC3^+^ cells (Figures 7C–E). Quantification revealed that indeed apical BrdU^+^ cells were more often apoptotic while EdU^+^ cells were not. Since apical active microglia were detected more often in the hyperoxia group, we finally investigated whether there are changes in elimination of apoptotic cells by Iba1^+^/CD68^+^ active microglia. However, we have neither found any increase in the number of active microglia-targeting CC3^+^ cells nor in the number of apoptotic cells that were engulfed by microglia in hyperoxic brain when compared to controls (Figures 7F–H).

### Number of Excitatory Synapses Follows Normalization in L5

**FIGURE 8:**
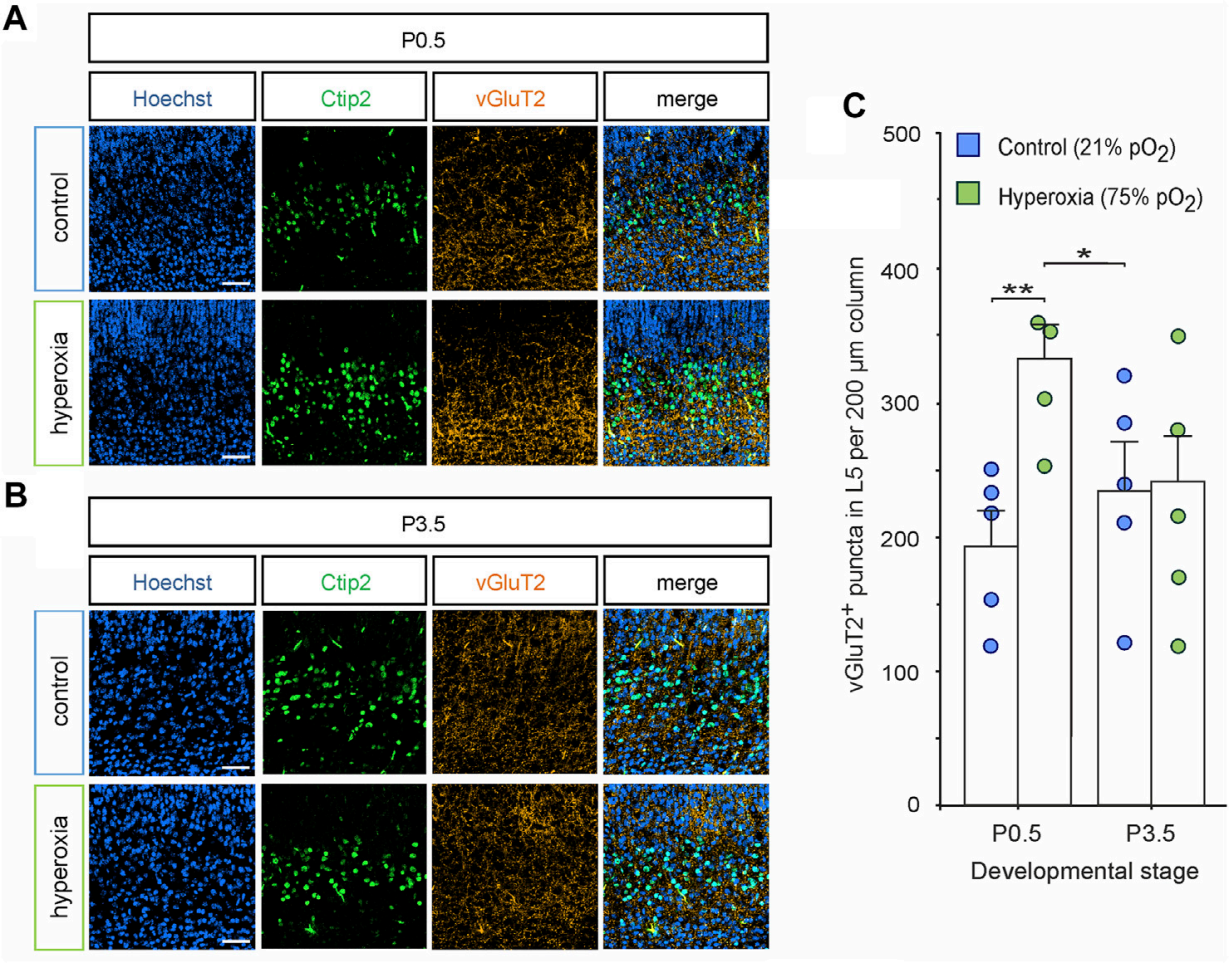
Synaptic excitatory input into L5 is normalized together with L5 neurons. **(A,B)** Representative fluorescent images of vGluT2^+^ synapses (orange) in L5 (Ctip2^+^ cells, green) in the middle cortical sections along the rostro-caudal axis of hyperoxia-treated and control mice at P0.5 **(A)** and P3.5 **(B)**. Hoechst (blue) was used to stain cell nuclei. Scale bars represent 50 µm. **(C)** Quantification of vGluT2^+^ puncta showed an increased number of puncta at P0.5, but not at P3.5. Data are means ± s.e.m. (*n* = 5 for all animal groups). *p < 0.05 and **p < 0.01 from two-way ANOVA with *post-hoc* two-sided *t*-test (non-significant comparisons are not marked for clarity). For full statistics, see Supplementary Table S10.

To provide first data on the effects on mid-neurogenesis hyperoxygenation on synaptic development, we analyzed the expression of the vesicular glutamate transporter 2 (vGluT2) as a common target for microglia pruning in later stages and a marker for the predominant form of excitatory synapses during early brain development (Nakamura et al., 2007; Schafer et al., 2012) (Figure 8). Double staining of vGluT2 and Ctip2 showed increased synaptic input into L5 in the hyperoxia animal group at P0.5: quantification in L5 showed that there were 1.7-fold more vGluT2^+^ puncta in the hyperoxia group as compared with normoxic controls at P0.5 (*p* = 0.006), which did not persist until P3.5 (Figure 8C). Additionally, the number of vGluT2^+^ puncta in L5 in the hyperoxia group at P0.5 temporary overshots that of controls at P0.5 and P3.5 (*p* = 0.033).

## Discussion

We present here that the short-term effects of hyperoxygenation during mid-neurogenesis of fetal mouse brain development (E14.5 to E16.5) with increased neuroprecursor cell proliferation within the SVZ/OSVZ (Wagenfuhr et al., 2015) translate into an accelerated cortical development but without increase in cortical neurogenesis and cortical volume at early postnatal stage. Indeed, the CP is expanded through a specific overshoot amount of Ctip2^+^/Tbr1^−^ L5 neurons in later fetal development until birth in hyperoxic mouse cortex, which is normalized at early postnatal stage. This normalization is accompanied by an increase of microglial cells within L5 capable of targeting the respective neurons but no signs of L5 neuronal apoptosis.

We used our established model of maternal hyperoxygenation to investigate the effects of increased fetal brain oxygen tension during mid-neurogenesis (E14.5–E16.5) on later cortical development until the early postnatal state (Wagenfuhr et al., 2015; Wagenfuhr et al., 2016). Early chronic hyperoxygenation in this model causes severe reduction of neuroprecursor cell proliferation and the apical neuroprecursor cell pool (Markert et al., 2020). Contributing to this, Lange et al. (2016) reported that early hyperoxygenation from E10.5 to E13.5 is able to alter neuroprogenitor cell fate leading to a decrease of expanding neuroprogenitors. However, late or postnatal hyperoxygenation is known to cause brain damage accompanied by excessive loss of neurons (Gerstner et al., 2008; Yis et al., 2008; Tuzun et al., 2012), while short-term hyperoxygenation during mid-neurogenesis of fetal mouse brain development (E14.5 to E16.5) leads to an immediate expansion of a distinct proliferative cell population basal of the SVZ, which subsequently contributes to corticogenesis by heading for deeper cortical layers (Wagenfuhr et al., 2015). Finally, the amount of Ctip2^+^ neurons in L5 projecting into various brain regions is markedly overshot after short-term maternal hyperoxygenation and at birth with a normalization until early postnatal stage (Hattox and Nelson 2007; Oswald et al., 2013; Wagenfuhr et al., 2015). Oxygen levels are known to directly regulate neuroprecursor cell maintenance, proliferation, and differentiation *in vitro* through the activation of several oxygen-sensitive signaling pathways (Chen et al., 2007; Pistollato et al., 2007; Giese et al., 2010; Mazumdar et al., 2010; Braunschweig et al., 2015; Mennen et al., 2020). Although the *in vitro* cell models are not directly comparable to our *in vivo* system, analyses of the fetal brain oxygen tension revealed that maternal hyperoxygenation of 75% leads to an increase of oxygen tension in the neurogenic niche of the VZ/SVZ from below 1.1% in maternal normoxic condition to oxygen levels above this threshold in hyperoxic animals, which is also supported by other colleagues (Wagenfuhr et al., 2015; Lange et al., 2016). Of note, there are no *in vivo* oxygen markers available for small laboratory animals to detect changes in oxygen levels in the range of 5%–20% to further define the tissue oxygen tension in the hyperoxic condition. However, maternal hyperoxia is unlikely to cause oxygen levels towards 20%, which is the commonly used oxygen tension in cell culture experiments. Thus, the higher oxygen tension in hyperoxic brain tissue likely represents the *in vitro* condition of mild hyperoxia and provides a stimulating environment for maintenance and proliferation of neuroprecursor cells as demonstrated in cell culture (Chen et al., 2007; Chen et al., 2010; Santilli et al., 2010; Braunschweig et al., 2015; Qi et al., 2017) when compared to very low oxygen conditions.

The observed normalization of the brain morphology in the early postnatal stage indicates that the brain is able to compensate prenatally evoked morphological changes at least for an excess of cell population. Within the period of synaptogenesis during the first 30 postnatal days, neuronal programmed cell death or apoptosis is known to play a major role in shaping the neocortex with a peak around P5 in rodents in most studies (Southwell et al., 2012; Ahern et al., 2013; Blanquie et al., 2017). In the six-layered isocortex, the loss of neuronal density displays a layer-specific pattern and manifested itself mostly in L2–L4, whereas L1, L5, and L6 show fewer changes. We thus studied whether this physiological process is also mediating the normalization neuronal cell counts in L5 of hyperoxic mouse cortex in earlier postnatal stages by using CC3 staining (Fernandes-Alnemri et al., 1994; Nicholson et al., 1995; Wong et al., 2018). Surprisingly, almost none of L5 Ctip2^+^ neurons showed CC3 marker expression, indicating that apoptosis is not involved in controlling the number of L5 neurons after mid-neurogenesis hyperoxygenation. The rare occurrence of apoptosis in L5 in the first days after birth is however in line with previous systematic studies of layer-specific postnatal apoptosis (Verney et al., 2000; Denaxa et al., 2018). However, we found an increase in the number of CC3^+^ apoptotic cells in hyperoxic as compared to normoxic brain at P0.5, but their location was vastly limited to the apical proliferative zone outside the CP, and they were eliminated by active microglia. This phenomenon might be interpreted as an adaptive response of the physiological apical apoptosis at birth to the hyperoxia-induced increased proliferation of precursor cells at E14.5 and at the end of mid-neurogenesis phase at E16.5 (White and Barone 2001; Wagenfuhr et al., 2015). Putative feedback mechanisms regulating neuroprecursor cell proliferation such as activity-dependent negative feedback from developing neurons (Toma et al., 2014) might be involved in cortical re-shaping after mid-neurogenesis hyperoxygenation. We thus applied the thymidine analogue EdU at a time point when it is known that the brain enlarges (E17.5) and 1 day after the oxygen treatment and analyzed EdU uptake at birth (P0.5). Although hyperoxygenation provokes strong immediate effects on the number of CP neurons born at E14.5 as described earlier (Wagenfuhr et al., 2015), cells generated after the hyperoxygenation phase at E17.5 predominantly reside on the apical side of the developing cortex and do not yet contribute to CP morphology at birth. Moreover, there is no indication for an adaption regarding the number of neurons in the CP during the post-hyperoxygenation phase.

Microglia already physiologically colonize the developing brain at E10.5 where they regulate the number of precursor cells through phagocytosis (Cunningham et al., 2013; Arnò et al., 2014; Tronnes et al., 2016). Until E16.5, microglia exclusively reside within the proliferating zones while they start to invade into the CP as late as during the early postnatal stage (Squarzoni et al., 2014). The majority of microglia in the developing cerebral cortex have an activated morphology and express markers associated with activation, and functional studies revealed that microglia regulate neuroprecursor cell number in the developing cortex by phagocytosis (Cunningham et al., 2013). Interestingly, most precursor cells targeted by Iba1^+^ microglia in the cortical proliferative zones did not show signs of cell death or apoptosis (Fricker et al., 2012; Cunningham et al., 2013). Our data suggest a very similar mechanism during the re-shaping of the cortical layers after mid-neurogenesis hyperoxygenation: increased numbers of Iba1^+^ microglia, which also express CD68 as a marker for active microglia (Rabinowitz and Gordon 1991; Jurga et al., 2020), target and incorporate Ctip2^+^ L5 neurons with no apoptotic signs at the critical stage P0.5. However, the number of targeted Satb2^+^ cells or incorporated Satb2^+^ particles at the same time remains unaffected, suggesting a specific effect on Ctip2^+^ L5 neurons. Nevertheless, our study is limited to immunohistochemical staining and cannot exclude other effects such as a critical change in microglial support for surviving of L5 cells (Ueno et al., 2013). In addition, the study is limited to a rather small sample size. Non-significant trends like the increased number of microglia in SP/L6 at E16.5 could potentially be relevant. Since there are no changes in microglial activity or absolute L6-specific Tbr1^+^ neurons, this may reflect an accelerated invasion of the cortical plate where microglia migrate from apical through SP and L6 to L5 (Swinnen et al., 2013). Consequently, future functional studies with activation and depletion of microglia during late prenatal and early postnatal cortical development are warranted to investigate the exact microglia–L5 neuron interactions in cortical re-shaping after critical insults during mid-neurogenesis such as hyperoxygenation. These data in conjunction with no indications for changes in cell migration [data on cortical layering herein and Wagenfuhr et al., (2015)] or compensatory reduction or shift of precursor proliferation strongly suggest different mechanisms to normalize the overshoot amount of neuroprecursor cells depending on the brain region with CC3-mediated apoptosis as one major mechanism within the apical proliferative zone (VZ/SVZ) and microglia playing a key role in cortical L5.

To first shed light on alterations of early synaptic connectivity within the developing cortex by mid-neurogenesis hyperoxygenation, we further analyzed the expression of vGluT2 as a common marker for the predominant form of excitatory synapses during early brain development (Nakamura et al., 2007). We detected a temporary overshoot of glutamatergic synaptic input into L5 in the hyperoxia animal group at P0.5 with normalization until P3.5 in fairly accurate parallelism to the changes in cortical L5 neurogenesis. Although the underlying mechanisms of this normalization need to be determined by functional studies as outlined above, it might also be mediated through activated microglia, because microglia have been reported to have a pivotal role in remodeling of developing synapses in the early postnatal brain (Schafer et al., 2012). To determine whether the morphological changes in response to hyperoxygenation during mid-neurogenesis translate into behavioral disruption, early postnatal behavioral testing of the pups using righting reflex test, gait analysis, and negative geotaxis test (Lubics et al., 2005; Fan et al., 2008) is urgently required in future studies. Our observations might then be of interest for investigating layer 5-specific neurodevelopmental disorders (Kolomeets and Uranova 2019; Mi et al., 2019) and their potential therapeutic/prophylactic interventions.

Together, the present data demonstrate that fetal brain hyperoxygenation during mid-neurogenesis from embryonic stage E14.5 to E16.5 accelerates cortical development in the fetal mouse brain. The cortical CP is expanded through a specific overshoot amount of L5 neurons at E16.5 and at birth in hyperoxic mouse cortex, which is subsequently normalized at early postnatal stage. This normalization is accompanied by an increase of microglial cells within L5 capable of targeting and incorporating the respective neurons with no signs of L5 neuronal apoptosis. Indeed, our data strongly suggest different mechanisms to the overshoot number of neuroprogenitor cells depending on the brain region with CC3-mediated apoptosis as the mechanism within the apical proliferative zone and microglial targeting in cortical L5. However, future functional studies on microglia using ablation and/or stimulation of microglia are warranted to finally confirm that an increased microgliosis in L5 is responsible or at least contribute to postnatal adaption to prenatal hyperoxia effects on corticogenesis.

## Data Availability

The raw data supporting the conclusion of this article will be made available by the authors, without undue reservation.

## Abbreviations

CC3, cleaved caspase 3; CP, cortical plate; Ctip2, chicken ovalbumin upstream promotor transcription factor-interacting protein 2; E, embryonic day; L, layer; NeuN, neuronal nuclear protein; RBFOX3, RNA binding protein fox-1 homolog 3; OSVZ, outer subventricular zone; P, postnatal stage; Pax6, paired box 6; PBS, phosphate buffered saline; Satb2, special AT-rich sequence-binding protein 2; SVZ, subventricular zone; Tbr1, T-box brain protein 1; Tbr2, T-box brain protein 2; TBST, Tris buffered saline/Tween 20; vGluT2, vesicular glutamate transporter 2; VZ, ventricular zone.
