# Supplementary material for: Hyperoxygenation During Mid-Neurogenesis Accelerates Cortical Development in the Fetal Mouse Brain
Source: Front Cell Dev Biol. 2022 Mar 17;10:732682. doi: 10.3389/fcell.2022.732682 (PMC8969024; doi:10.3389/fcell.2022.732682)
Supplement: Supplementary file 1 [file DataSheet1.pdf]

Hyperoxygenation during mid-neurogenesis accelerates cortical development in the fetal mouse brain

Franz Markert and Alexander Storch

Supplementary Figures:

- **Supplementary Figure S1.** Example for the determination of the volume of the cortical plate.
- **Supplementary Figure S2.** Effects of maternal hyperoxygenation on the absolute number of layer specific neurons.
- **Supplementary Figure S3.** Effects of maternal hyperoxygenation on the distribution of microglia in a P16.5 and P3.5 mouse cortex.
- **Supplementary Figure S4.** Effects of hyperoxygenation on the total number of microglia within the developing cortex.
- **Supplementary Figure S5.** Iba1$^+$ cells are able to target Satb2$^+$ cells.

Supplementary Tables:

- **Supplementary Table S1.** Statistics determined for NeuN$^+$ cortical neurons.
- **Supplementary Table S2.** Statistics determined for Tbr1$^+$ cortical neurons.
- **Supplementary Table S3.** Statistics determined for Ctip$^+$/Tbr1$^-$neurons.
- **Supplementary Table S4.** Statistics determined for Satb2$^+$ cortical neurons.
- **Supplementary Table S5.** Statistics determined for apical Iba1$^+$ cells.
- **Supplementary Table S6.** Statistics determined for subplate/layer 6 (SP/L6) Iba1$^+$ cells.
- **Supplementary Table S7.** Statistics determined for layer 5 (L5) Iba1$^+$ cells
- **Supplementary Table S8.** Statistics determined for layer 4-1 (L4-1) Iba1$^+$ cells.
- **Supplementary Table S9.** Statistics determined for absolute CC3$^+$ cell counts.
- **Supplementary Table S10.** Statistics determined for vGluT2$^+$ synapses in L5.
- **Supplementary Table S11.** Statistics determined for absolute Tbr1$^+$ neuron counts.
- **Supplementary Table S12.** Statistics determined for absolute Ctip$^+$/Tbr1$^-$ neuron counts.
- **Supplementary Table S13.** Statistics determined for absolute Satb2$^+$ neuron counts.
- **Supplementary Table S14.** Statistics determined for total Iba1$^+$ cell counts.

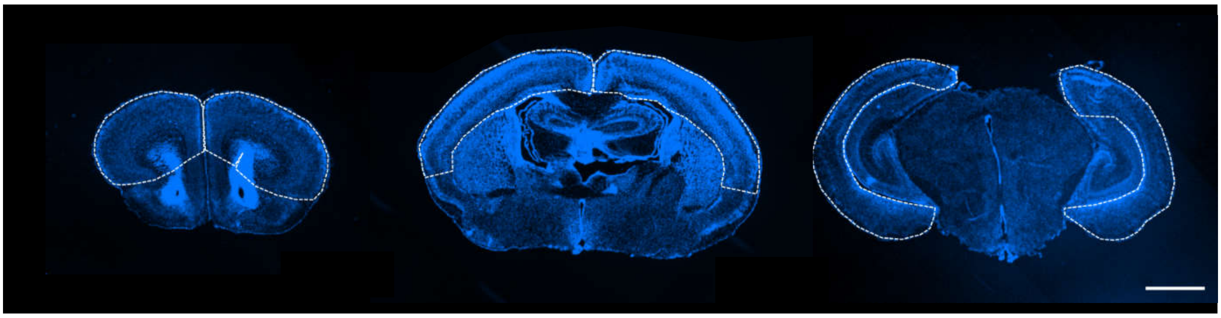

Supplementary Figure S1. Example for the determination of the volume of the cortical plate (CP). Every 6th Hoechst stained slice of a mouse brain was outlined as shown in the figure (left to right: rostral, middle and caudal section) and used to calculate the volume. Scale bar, 1000 µm.

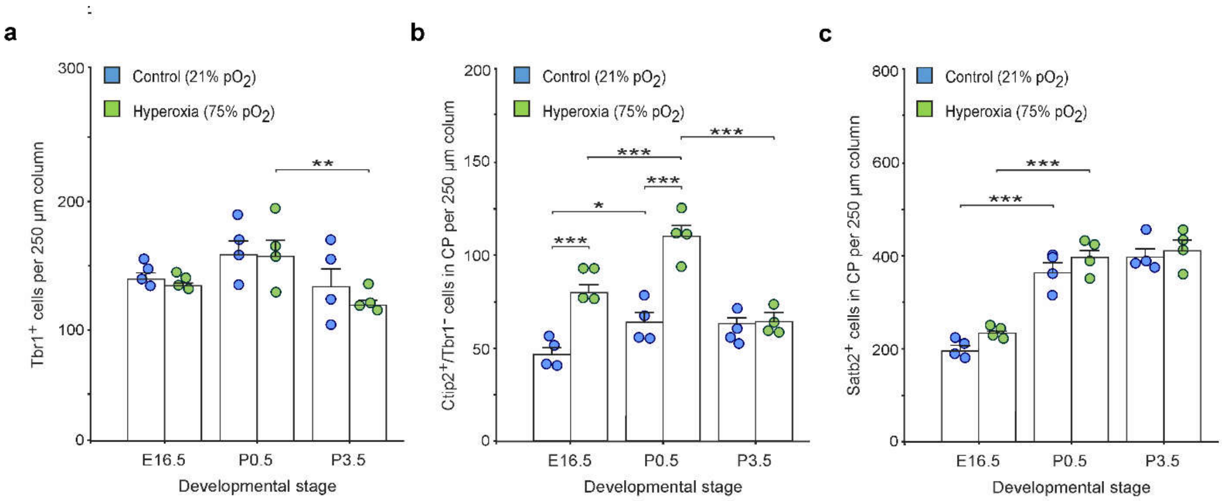

Supplementary Figure S2. Effects of maternal hyperoxygenation on the absolute number of layer specific neurons. Quantification of absolute Tbr1^{+}, Ctip2^{+}/Tbr1^{-} and Satb2^{+} cells within 250 µm wide cortical columns of E16.5, P0.5 and P3.5 mice. Data are means±s.e.m. (n = 4). * *p*<0.05, ** *p*<0.01, *** *p*<0.001 from two-way ANOVA with *post-hoc* two-sided t-test with Bonferroni correction. For full statistics, see **Supplementary Tables S11-S13**.

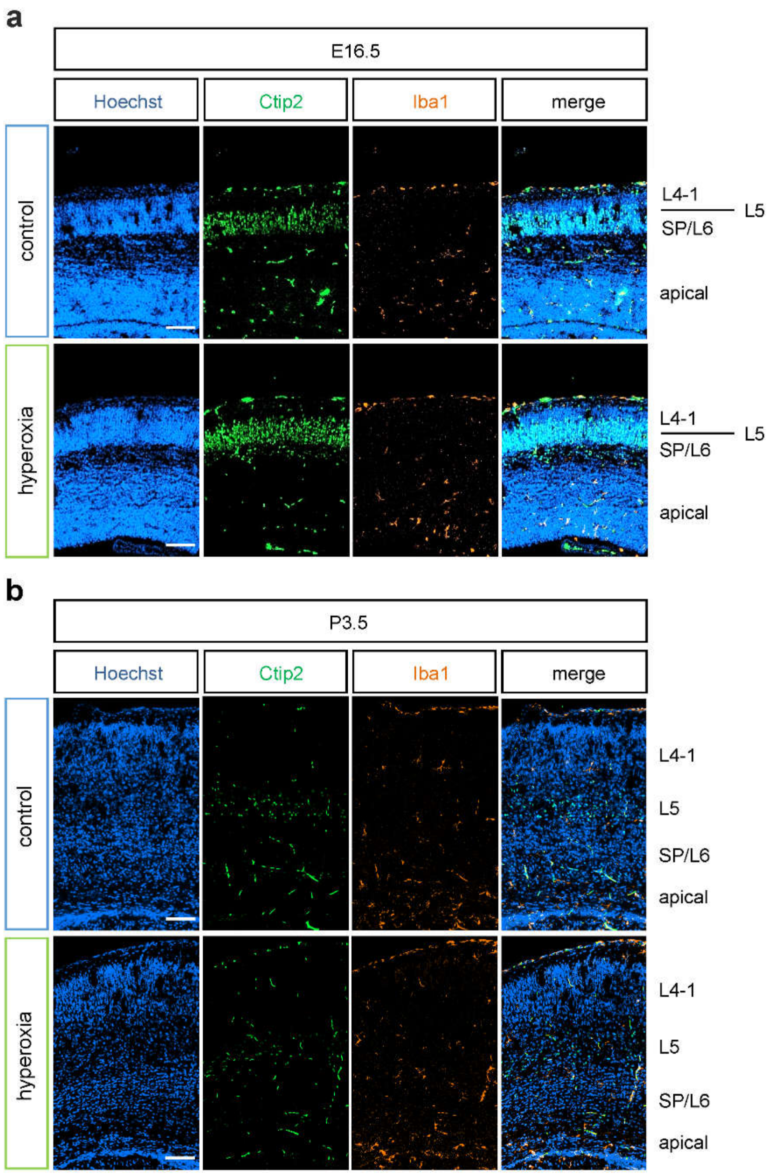

Supplementary Figure S3: Effects of fetal brain hyperoxygenation on the distribution of microglia in E16.5 and P3.5 mouse cortex. Representative fluorescent images of Iba1^{+} cells (orange) from (**a**) E16.5 and (**b**) P3.5 in the middle cortical sections along the rostro-caudal axis–of hyperoxia treated and control mice showed no differences. Ctip2^{+} (green) was used for layer determination and Hoechst (blue) was used to stain cell nuclei. Scale bars represent 100 µm.

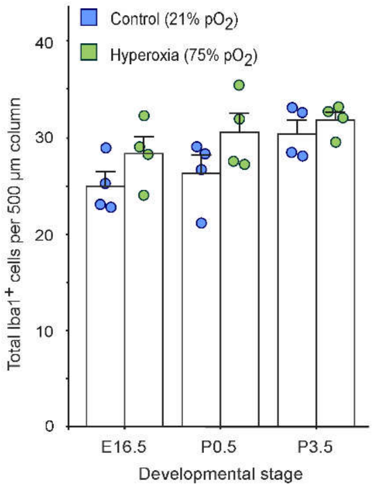

Supplementary Figure S4. Effects of hyperoxygenation on the total number of microglia within the developing cortex. Quantification of the total number of $Iba1^{+}$ microglia showed no differences with respect to hyperoxia treatment. Data are means±s.e.m. (n = 4). * $p<0.05$, ** $p<0.01$, *** $p<0.001$ from two-way ANOVA with *post-hoc* two-sided t-test with Bonferroni correction. For full statistics, see **Supplementary Table S14.**

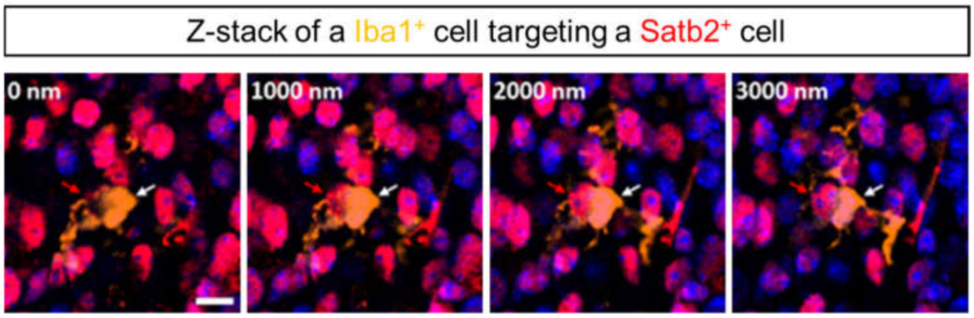

Supplementary Figure S5. $Iba1^{+}$ cells are able to target $Satb2^{+}$ cells. Representative z-stack images of a microglia cell (white arrow) targeting $Satb2^{+}$ cells (red arrow) in a P0.5 mouse cortex. Scale bars represent 10 µm.

Supplementary Tables

Supplementary Table S1. Statistics determined for NeuN$^+$ cortical neurons in various development stages (E16.5, P0.5, P3.5) after different oxygen exposures during mid-neurogenesis (**Figure 1e**). Two-way ANOVA with *post-hoc* t-test and Bonferroni adjustment with atmospheric oxygen concentrations and development stage as fixed factors revealed that atmospheric oxygen concentration and developmental stage have a significant interaction effect on NeuN$^+$ neuron counts (p=0.028, F-value=4.2) and significant differences among atmospheric oxygen concentrations (p=0.006, F-value=8.8) and developmental stages (p<0.001, F-value=9.3). Displayed are Bonferroni-adjusted P-values (E16.5: n=4 [control], n=3 [hyperoxia]; P0.5: n=8 [control], n=6 [hyperoxia]; P3.5: n=4 [control], n=6 [hyperoxia]). **(A)** Significances among the different atmospheric oxygen concentrations. **(B)** Significances among the different developmental stages. Bold values indicate significant differences.

A

	**E16.5**	**P0.5**	**P3.5**
**Normoxia (21% O_2) vs. Hyperoxia (75% O_2)**	**0.005**	**0.013**	0.581

B

	**Normoxia (21% O_2))**	**Hyperoxia (75% O_2)**
**E16.5 vs. P0.5**	**0.0459**	1
**E16.5 vs. P3.5**	**< 0.001**	0,769
**P0.5 vs. P3.5**	**0.011**	1

Supplementary Table S2. Statistics determined for Tbr1^{+} cortical neurons in various development stages (E16.5, P0.5, P3.5) after different oxygen exposures during mid-neurogenesis (**Figure 2b**). Two-way ANOVA with *post-hoc* t-test and Bonferroni adjustment with atmospheric oxygen concentrations and development stage as fixed factors revealed that atmospheric oxygen concentration and developmental stage have no significant interaction effect on Tbr1^{+} neuron counts (p=0.210, F-value=1.7), but significant differences among atmospheric oxygen concentrations (p=0.006, F-value=9.8) and developmental stages (p<0.001, F-value=84.3). Displayed are Bonferroni-adjusted P-values (n = 4). **(A)** Significances among the different atmospheric oxygen concentrations. **(B)** Significances among the different developmental stages. Bold values indicate significant differences.

A

	**E16.5**	**P0.5**	**P3.5**
**Normoxia (21% O_2) vs. Hyperoxia (75% O_2)**	**0.004**	0.333	0.278

B

	**Normoxia (21% O_2)**	**Hyperoxia (75% O_2)**
**E16.5 vs. P0.5**	**< 0.001**	**0.002**
**E16.5 vs. P3.5**	**< 0.001**	**< 0.001**
**P0.5 vs. P3.5**	**0.005**	0.004

Supplementary Table S3. Statistics determined for $Ctip^+/Tbr1^-$ cortical neurons in various development stages (E16.5, P0.5, P3.5) after different oxygen exposures during mid-neurogenesis (**Figure 2c**). Two-way ANOVA with *post-hoc* t-test and Bonferroni adjustment with atmospheric oxygen concentrations and development stage as fixed factors revealed that atmospheric oxygen concentration and developmental stage have a significant interaction effect on $Ctip^+/Tbr1^-$ cortical neuron counts (p=0.002, F-value=9.5) and significant differences among atmospheric oxygen concentrations (p<0.001, F-value=49.2) and developmental stages (p=0.002, F-value=44.9). Displayed are Bonferroni-adjusted P-values (n = 4). **(A)** Significances among the different atmospheric oxygen concentrations. **(B)** Significances among the different developmental stages. Bold values indicate significant differences.

A

	**E16.5**	**P0.5**	**P3.5**
**Normoxia (21% O_2) vs. Hyperoxia (75% O_2)**	**< 0.001**	**< 0.001**	0.629

B

	**Normoxia (21% O_2))**	**Hyperoxia (75% O_2)**
**E16.5 vs. P0.5**	0.135	0.092
**E16.5 vs. P3.5**	**0.003**	**< 0.001**
**P0.5 vs. P3.5**	0.309	**< 0.001**

Supplementary Table S4. Statistics determined for Satb2^{+} cortical neurons in various development stages (E16.5, P0.5, P3.5) after different oxygen exposures during mid-neurogenesis (**Figure 2d**). Two-way ANOVA with *post-hoc* t-test and Bonferroni adjustment (n = 4) with atmospheric oxygen concentrations and development stage as fixed factors revealed that atmospheric oxygen concentration and developmental stage have no significant interaction effect on Satb2^{+} neuron counts (p=0.922, F-value=0.1), no significant differences among atmospheric oxygen concentrations (p=0.922, F-value=3.6), but significant differences among developmental stages (p=0.048, F-value=3.6). Displayed are Bonferroni-adjusted *P*-values (n = 4). **(A)** Significances among the different atmospheric oxygen concentrations. **(B)** Significances among the different developmental stages. Bold values indicate significant differences.

A

	**E16.5**	**P0.5**	**P3.5**
**Normoxia (21% O_2) vs. Hyperoxia (75% O_2)**	0.411	0.748	0.437

B

	**Normoxia (21% O_2)**	**Hyperoxia (75% O_2)**
**E16.5 vs. P0.5**	0.141	0.369
**E16.5 vs. P3.5**	0.720	0.774
**P0.5 vs. P3.5**	1	1

Supplementary Table S5. Statistics determined for apical Iba1^{+} cells in various development stages (E16.5, P0.5, P3.5) after different oxygen exposures during mid-neurogenesis (**Figure 4b**). Two-way ANOVA with *post-hoc* t-test and Bonferroni adjustment with atmospheric oxygen concentrations and development stage as fixed factors revealed that atmospheric oxygen concentration and developmental stage have no significant interaction effect on apical Iba1^{+} cell counts (p=0.465, F-value=0.8) and no significant differences among atmospheric oxygen concentrations (p=0.363, F-value=0.9), but significant differences among developmental stages (p<0.001, F-value=20.9). Displayed are Bonferroni-adjusted P-values (n = 4). **(A)** Significances among the different atmospheric oxygen concentrations. **(B)** Significances among the different developmental stages. Bold values indicate significant differences.

A

	**E16.5**	**P0.5**	**P3.5**
**Normoxia (21% O_2) vs. Hyperoxia (75% O_2)**	0.146	0.819	0.744

B

	**Normoxia (21% O_2))**	**Hyperoxia (75% O_2)**
**E16.5 vs. P0.5**	0.605	**0.020**
**E16.5 vs. P3.5**	**0.003**	**< 0.001**
**P0.5 vs. P3.5**	**0.049**	0.064

Supplementary Table S6. Statistics determined for subplate/layer 6 (SP/L6) Iba1^{+} cells in various development stages (E16.5, P0.5, P3.5) after different oxygen exposures during mid-neurogenesis (**Figure 4c**). Two-way ANOVA with *post-hoc* t-test and Bonferroni adjustment with atmospheric oxygen concentrations and development stage as fixed factors revealed that atmospheric oxygen concentration and developmental stage have no significant interaction effect on SP/L6 Iba1^{+} cells counts (p=0.295, F-value=1.3) and no significant differences among atmospheric oxygen concentrations (p=0.203, F-value=1.7), but significant differences among developmental stages (p<0.001, F-value=38.6). Displayed are Bonferroni-adjusted P-values (n = 4). **(A)** Significances among the different atmospheric oxygen concentrations. **(B)** Significances among the different developmental stages. Bold values indicate significant differences.

A

	**E16.5**	**P0.5**	**P3.5**
**Normoxia (21% O_2) vs. Hyperoxia (75% O_2)**	0.942	0.054	0.772

B

	**Normoxia (21% O_2)**	**Hyperoxia (75% O_2)**
**E16.5 vs. P0.5**	0.096	**< 0.001**
**E16.5 vs. P3.5**	**< 0.001**	**< 0.001**
**P0.5 vs. P3.5**	**0.005**	0.211

Supplementary Table S7. Statistics determined for layer 5 (L5) Iba1$^+$ cells in various development stages (P0.5, P3.5) after different oxygen exposures during mid-neurogenesis (**Figure 4d**). Two-way ANOVA with *post-hoc* t-test and Bonferroni adjustment with atmospheric oxygen concentrations and development stage as fixed factors revealed that atmospheric oxygen concentration and developmental stage have a significant interaction effect on L5 Iba1$^+$ cells counts (p=0.014, F-value=8.1) and significant differences among atmospheric oxygen concentrations (p=0.003, F-value=13.2) and developmental stages (p<0.001, F-value=37.5). Displayed are Bonferroni-adjusted P-values (n = 4). **(A)** Significances among the different atmospheric oxygen concentrations. **(B)** Significances among the different developmental stages. Bold values indicate significant differences.

A

	**P0.5**	**P3.5**
**Normoxia (21% O_2) vs. Hyperoxia (75% O_2)**	**< 0.001**	0.588

B

	**Normoxia (21% O_2))**	**Hyperoxia (75% O_2)**
**P0.5 vs. P3.5**	**< 0.001**	**0.039**

Supplementary Table S8. Statistics determined for layer 4-1 (L4-1) Iba1$^+$ cells in various development stages (P0.5, P3.5) after different oxygen exposures during mid-neurogenesis (**Figure 4e**). Two-way ANOVA with *post-hoc* t-test and Bonferroni adjustment with atmospheric oxygen concentrations and development stage as fixed factors revealed that atmospheric oxygen concentration and developmental stage have no significant interaction effect on L4-1 Iba1$^+$ cells counts (p=0.945, F-value=0.0) and no significant differences among atmospheric oxygen concentrations (p=0.945, F-value=0.0), but significant differences among developmental stages (p<0.001, F-value=85.8). Displayed are Bonferroni-adjusted P-values (n = 4). **(A)** Significances among the different atmospheric oxygen concentrations. **(B)** Significances among the different developmental stages. Bold values indicate significant differences.

A

	**P0.5**	**P3.5**
**Normoxia (21% O_2) vs. Hyperoxia (75% O_2)**	0.920	1.000

B

	**Normoxia (21% O_2)**	**Hyperoxia (75% O_2)**
**P0.5 vs. P3.5**	**< 0.001**	**< 0.001**

Supplementary Table S9. Statistics determined for $CC3^+$ cell counts in various development stages (E16.5, P0.5, P3.5) after different oxygen exposures during mid-neurogenesis (**Figure 7b**). Robust ANOVA using raov function from Rfit package with *post-hoc* unpaired Wilcoxon-test and Bonferroni adjustment (n = 3) with atmospheric oxygen concentrations and development stage as fixed factors revealed that atmospheric oxygen concentration and developmental stage have a significant interaction effect on $CC3^+$ cell counts (p=0.001, F-value=9.1) and significant differences among atmospheric oxygen concentrations (p=0.004, F-value=10.4) and developmental stages (p<0.001, F-value=30.1). **(A)** Significances among the different atmospheric oxygen concentrations. **(B)** Significances among the different developmental stages. Bold values indicate significant differences.

A

	**E16.5**	**P0.5**	**P3.5**
**Normoxia (21% O_2) vs. Hyperoxia (75% O_2)**	0.564	**0.008**	1.000

B

	**Normoxia (21% O_2))**	**Hyperoxia (75% O_2)**
**E16.5 vs. P0.5**	**0.014**	**0.024**
**E16.5 vs. P3.5**	0.075	0.107
**P0.5 vs. P3.5**	0.276	0.786

Supplementary Table S10: Statistics determined for vGluT2^{+} synapses in L5 (P0.5, P3.5) after different oxygen exposures during mid-neurogenesis (**Figure 8**). Two-way ANOVA with *post-hoc* t-test with atmospheric oxygen concentrations and development stage as fixed factors revealed that atmospheric oxygen concentration and developmental stage have a significant interaction effect on VGlut2^{+} synapses (*p*=0.046, F-value=4.7) and significant differences among atmospheric oxygen concentrations (*p*=0.030, F-value=5.6), but no significant differences among developmental stages (*p*=0.371, F-value=0.8). Displayed are *P*-values (n = 5). **(A)** Significances among the different atmospheric oxygen concentrations. **(B)** Significances among the different developmental stages. Bold values indicate significant differences.

A

	**P0.5**	**P3.5**
**Normoxia (21% O_2) vs. Hyperoxia (75% O_2)**	**0.006**	0.881

B

	**Normoxia (21% O_2))**	**Hyperoxia (75% O_2)**
**P0.5 vs. P3.5**	0.394	**0.045**

Supplementary Table S11. Statistics determined for absolute Tbr1^{+} cortical neuron counts in various development stages (E16.5, P0.5, P3.5) after different oxygen exposures during mid-neurogenesis (**Supplementary Figure S2a**). Two-way ANOVA with *post-hoc* t-test and Bonferroni adjustment with atmospheric oxygen concentrations and development stage as fixed factors revealed that atmospheric oxygen concentration and developmental stage have no significant interaction effect on Tbr1^{+} neuron counts (p=0.780, F-value=0.3) and no significant differences among atmospheric oxygen concentrations (p=0.338, F-value=1.0), but significant differences among developmental stages (p=0.014, F-value=5.5). Displayed are Bonferroni-adjusted P-values (n = 4). **(A)** Significances among the different atmospheric oxygen concentrations. **(B)** Significances among the different developmental stages. Bold values indicate significant differences.

A

	**E16.5**	**P0.5**	**P3.5**
**Normoxia (21% O_2) vs. Hyperoxia (75% O_2)**	0.654	0.895	0.278

B

	**Normoxia (21% O_2))**	**Hyperoxia (75% O_2)**
**E16.5 vs. P0.5**	0.567	0.327
**E16.5 vs. P3.5**	1.000	0.852
**P0.5 vs. P3.5**	0.261	**0.036**

Supplementary Table S12. Statistics determined for absolute Ctip2$^+$/Tbr1$^-$neuron counts in various development stages (E16.5, P0.5, P3.5) after different oxygen exposures during mid-neurogenesis (**Supplementary Figure S2b**). Two-way ANOVA with *post-hoc* t-test and Bonferroni adjustment with atmospheric oxygen concentrations and development stage as fixed factors revealed that atmospheric oxygen concentration and developmental stage have a significant interaction effect on Ctip2$^+$/Tbr1$^-$ neuron counts (p<0.001, F-value=10.8) and significant differences among atmospheric oxygen concentrations (p<0.001, F-value=53.2) and developmental stages (p<0.001, F-value=19.1). Displayed are Bonferroni-adjusted P-values (n = 4). **(A)** Significances among the different atmospheric oxygen concentrations. **(B)** Significances among the different developmental stages. Bold values indicate significant differences.

A

	**E16.5**	**P0.5**	**P3.5**
**Normoxia (21% O_2) vs. Hyperoxia (75% O_2)**	**< 0.001**	**< 0.001**	0.574

B

	**Normoxia (21% O_2))**	**Hyperoxia (75% O_2)**
**E16.5 vs. P0.5**	**0.047**	**< 0.001**
**E16.5 vs. P3.5**	0.208	0.057
**P0.5 vs. P3.5**	1.000	**< 0.001**

Supplementary Table S13. Statistics determined for absolute Satb2$^+$ cortical neuron counts in various development stages (E16.5, P0.5, P3.5) after different oxygen exposures during mid-neurogenesis (**Supplementary Figure S2c**). Two-way ANOVA with *post-hoc* t-test and Bonferroni adjustment (n = 4) with atmospheric oxygen concentrations and development stage as fixed factors revealed that atmospheric oxygen concentration and developmental stage have no significant interaction effect on Satb2$^+$ neuron counts (p=0.828, F-value=0.2), no significant differences among atmospheric oxygen concentrations (p=0.066, F-value=3.8), but significant differences among developmental stages (p<0.001, F-value=76.9). **(A)** Significances among the different atmospheric oxygen concentrations. **(B)** Significances among the different developmental stages. Bold values indicate significant differences.

A

	**E16.5**	**P0.5**	**P3.5**
**Normoxia (21% O_2) vs. Hyperoxia (75% O_2)**	0.153	0.225	0.527

B

	**Normoxia (21% O_2))**	**Hyperoxia (75% O_2)**
**E16.5 vs. P0.5**	**< 0.001**	**< 0.001**
**E16.5 vs. P3.5**	**< 0.001**	**< 0.001**
**P0.5 vs. P3.5**	0.573	1.000

Supplementary Table S14. Statistics determined for total Iba1$^+$ cell counts (E16.5, P0.5, P3.5) after different oxygen exposures during mid-neurogenesis (**Supplementary Figure S4**). Two-way ANOVA with *post-hoc* t-test and Bonferroni adjustment with atmospheric oxygen concentrations and development stage as fixed factors revealed that atmospheric oxygen concentration and developmental stage have no significant interaction effect on total Iba1$^+$ cell counts (p=0.643, F-value=0.5), but significant differences among atmospheric oxygen concentrations (p=0.030, F-value=5.5) and significant differences among developmental stages (p=0.034, F-value=4.1). Displayed are Bonferroni-adjusted P-values (n = 4). Significances among the different developmental stages. Bold values indicate significant differences.

A

	**E16.5**	**P0.5**	**P3.5**
**Normoxia (21% O_2) vs. Hyperoxia (75% O_2)**	0.144	0.069	0.541

B

	**Normoxia (21% O_2))**	**Hyperoxia (75% O_2)**
**E16.5 vs. P0.5**	1.000	0.989
**E16.5 vs. P3.5**	0.072	0.411
**P0.5 vs. P3.5**	0.234	1.000
